# From data to safer roads: predictive modelling and causal analysis of road fatalities in Australia

**DOI:** 10.1038/s41598-025-33744-7

**Published:** 2025-12-29

**Authors:** Saeid Afshari, Ali Soltani, Mohammad Amin Amiri

**Affiliations:** 1 Department of Computer Engineering, Shahreza Campus, University of Isfahan, Isfahan, Iran; 2 School of Humanities, Arts and Social Sciences (HASS), University of New England, Sydney, Australia; 3 Department of Computer Science, University of Tabriz, Tabriz, Iran

**Keywords:** Road safety, Fatality, Forecasting, Time-series analysis, Causal inference, Policy evaluation, Health care, Engineering

## Abstract

This study uses advanced time-series forecasting and causal modelling techniques to examine long-term patterns in Australian road traffic fatalities. Four statistical approaches were assessed: Holt-Winters, Theta, TBATS, and Vector Autoregression, with each offering strengths across different forecasting horizons. TBATS provided the most reliable short-term predictions, while Vector Autoregression performed best for medium- and long-term projections. A causal analysis using a random-effects panel model identified several key contributors to fatal crash risk, including older age groups, remote and outer-regional settings, nighttime periods, and high-speed environments. In contrast, younger adults and single-vehicle crashes were associated with lower fatality likelihood. Overall, the results demonstrate the value of flexible time-series techniques and panel data methods for guiding evidence-based road safety policy, targeted interventions, and infrastructure planning.

**Supplementary Information:**

The online version contains supplementary material available at 10.1038/s41598-025-33744-7.

## Introduction

Road traffic injuries represent one of the most enduring public health challenges in Australia. Despite advancements in vehicle technology, road design, and enforcement policies, the annual toll remains unacceptably high—with over 1100 fatalities recorded in recent years^1^. Over the past three decades, Australia implemented several major interventions, including random breath testing, mandatory seatbelt laws, safer road infrastructure, and vehicle safety improvements^2^. These reforms contributed to an overall downward trend in deaths and serious injuries, yet emerging threats such as mobile phone use, urban congestion, and climate-induced hazards continue to challenge progress^3^. Rural areas often face higher serious injury rates due to insufficient infrastructure and limited access to emergency services, while demographic factors such as age and gender influence crash risk due to differences in driving behavior and physical resilience^4^. Similarly, temporal factors, such as nighttime driving or seasonal variations, exacerbate crash severity under specific environmental conditions^5,6^. Investigating these variables through robust analytical techniques provides a multidimensional understanding of road safety, enabling targeted interventions that account for the unique risks faced by specific groups or regions^7,8^.

While recent research has explored integrating machine learning techniques with road safety analytics, the focus of this study is on advanced statistical modelling complemented by brief discussion of relevant machine learning considerations. Machine learning approaches can identify complex patterns in large datasets and have been applied to predict road traffic accidents and their severity by leveraging traffic, weather, and road condition data^9,10^. However, in this work the primary emphasis is on statistical frameworks. In particular, random-effects panel data modelling effectively captures both within- and between-entity variations, making it well suited for examining demographic and geographic influences over time^11^. Together with time-series forecasting models, such approaches provide interpretable and robust insights into road fatality trends, while also aligning with complementary evidence from machine learning studies^12^.

Although existing research has evaluated isolated risk factors—such as crash types, driver demographics, or regional disparities—there is a lack of integrated models that combine longitudinal forecasting with causal inference to capture the full dynamics of road fatalities. Studies have generally relied on either descriptive statistics (e.g.,^13^) or spatial analyses (e.g.,^14^) without coupling them with predictive modelling over extended timeframes. Furthermore, existing forecasting methods often ignore key temporal dependencies and lagged policy effects, which are essential for evaluating medium- and long-term intervention efficacy. This methodological gap hinders the development of proactive and evidence-driven safety strategies, particularly for vulnerable groups such as motorcyclists, pedal cyclists, pedestrians, and remote-area residents.

This study addresses this gap by applying a dual-method approach: (1) evaluating the forecasting performance of advanced time-series models (Holt-Winters, Theta, TBATS, and VAR), and (2) using a random-effects panel model to estimate the impact of geographic, demographic, and temporal factors on road fatalities. By integrating these methods, we aim to deliver both predictive accuracy and causal insight, enabling policymakers to target high-risk areas and populations with precision. The significance of this approach lies in its potential to inform future iterations of the National Road Safety Strategy (Office of Road Safety, 2020), support the design of Vision Zero policies, and guide infrastructure investments that are sensitive to spatiotemporal risk patterns. Moreover, the study contributes a replicable analytic framework for other jurisdictions seeking to blend machine-assisted forecasting with causal risk assessment. This research investigates two central questions:

- Forecasting Performance: How do different time-series forecasting models, including Holt-Winters, Theta, TBATS, and VAR perform in predicting road traffic fatalities in Australia, and which model is most effective for short-, mid-, and long-term horizons?
- Causal Risk Factors: What are the primary geographic, temporal, and demographic factors influencing crash fatalities in Australia, and how can these insights be translated into targeted, evidence-based road safety interventions?

Based on these research questions, we test the following five hypotheses:

Forecasting Hypotheses

H1: Advanced forecasting models (TBATS, VAR) would demonstrate superior accuracy (lower MAE, RMSE) in predicting Australian road traffic fatalities across short, mid, and long-term horizons compared to simpler models (Holt-Winters, Theta).

Causal Factor Hypotheses

- H2 (Geographic): Fatality rates will be significantly higher in major urban and remote/outer regional areas compared to other regions in Australia.
- H3 (Temporal): Summer months and nighttime driving periods will be associated with significantly increased road traffic fatalities.
- H4 (Demographic): Specific age groups (e.g., older adults 65 + , younger drivers in some contexts) will exhibit significantly higher fatality risks, while other age groups may show lower risks.
- H5 (Road/Policy Environment): Key Road environment characteristics (e.g., speed limit, crash location) will show statistically significant associations with the number of road fatalities.

### Research contribution

While previous studies have forecasted fatalities or identified risk factors, few have combined advanced multi-seasonal forecasting models like TBATS with robust causal inference techniques (VAR, Random-Effects) for a comprehensive, long-term analysis of Australian road fatalities, nor have they extensively explored the dynamic interplay of geographical, temporal, and demographic factors with the level of detail presented here. This study contributes by:

- Demonstrating the superior applicability of TBATS for long-term fatality forecasting in Australia, considering complex seasonality often overlooked.
- Employing VAR and Random-Effects models to provide nuanced causal insights into policy impacts, regional disparities, and vulnerable group risks, offering a dynamic and panel data perspective.
- Providing actionable, data-driven recommendations for Australian policymakers based on a rigorous, integrated analytical framework.

## Background studies

Road crashes are a leading cause of mortality and morbidity globally, with significant variability in outcomes influenced by a multitude of factors including crash characteristics, road user vulnerabilities, environmental conditions, and demographic profiles. Vulnerable road users, such as pedestrians, pedal cyclists, motorcyclists, children, and older adults, face disproportionate risks in road crashes due to their lack of physical protection and reduced resilience to injuries. Pedestrians and cyclists, for instance, are directly exposed to the impact of collisions without the protective infrastructure of a vehicle, making them particularly susceptible to severe injuries and fatalities^15^. Similarly, motorcyclists are at high risk due to limited protective gear and the inherent instability of motorcycles compared to four-wheeled vehicles. Age also plays a crucial role in determining fatality risks. Older adults often experience higher fatality rates due to declining physical resilience and slower reaction times, while children, with their smaller physiques, are less likely to survive high-impact collisions. Additionally, behavioral factors such as the use of mobile phones while walking or cycling, and environmental challenges such as inadequate pedestrian crossings and poorly designed urban infrastructure, exacerbate the vulnerability of these groups.

These disparities underline the need for targeted road safety interventions, including protective measures, urban infrastructure improvements, and public awareness campaigns tailored to the needs of vulnerable road users. In recent years, various studies have contributed to the understanding of traffic safety and accident dynamics. Fiorentini and Losa^16^ explored unobserved heterogeneity in factors contributing to fatal and injury crashes on Italian secondary road networks. Their research utilized both fixed and random parameters approaches to analyze the influences on crash occurrences, providing valuable insights for enhancing road safety measures. In the context of Ghana, Owusu-Ansah et al.^17^ focused on modeling road fatalities resulting from tricycle crashes in the Ashanti Region. By employing a regression model with ARIMA errors, they examined trends and patterns in tricycle-related fatalities, highlighting underlying causes and potential interventions to mitigate such incidents. Walker et al.^18^ investigated the severity of injuries from crashes occurring during winter weather conditions. Their study applied winter storm classification techniques to assess how varying types of winter weather impact crash injury severity, offering critical information for policymakers and emergency responders regarding winter road safety. Additionally, Alkaabi^19^ identified hotspot areas for traffic accidents while analyzing driver behaviors associated with these incidents. By employing various analytical techniques, this research elucidated the correlation between driver actions and accident occurrences, ultimately aiming to inform strategies for enhancing road safety and reducing traffic-related injuries. These studies collectively underscore the importance of understanding multifaceted factors influencing traffic accidents, thereby informing targeted interventions and policy decisions aimed at improving road safety.

The literature reveals a complex interplay between crash characteristics and road user types in determining road crash fatalities. Single-vehicle crashes are associated with significantly fewer fatalities, likely reflecting lower collision intensity compared to multi-vehicle incidents. On the other hand, crashes involving heavy rigid trucks are identified as major contributors to fatalities, emphasizing the severe consequences of high-impact collisions. These results underscore the necessity of targeted interventions, such as stricter regulations for heavy vehicle operation and enhanced crash avoidance systems^20^. The vulnerability of specific road user groups also stands out. Pedestrians and motorcyclists exhibit the highest fatality risks, corroborating prior studies on their exposure to harm due to a lack of physical protection^13^. In contrast, passengers show a reduction in fatalities, likely due to advancements in automotive safety technologies. These insights highlight the importance of continued innovation in protective measures for vulnerable road users, such as pedestrian airbags and advanced rider assistance systems. Additionally, education campaigns focusing on pedestrian safety and protective gear for motorcyclists are essential to mitigate these risks^21^. Temporal patterns demonstrate that evening and nighttime crashes elevate fatality risks, likely due to reduced visibility and impaired driving behaviors. Weekends and specific months, particularly February and March, also show higher fatality rates, potentially linked increased recreational travel and holiday-related risks^22^. These findings point to the critical need for temporal-specific interventions, such as enhanced lighting infrastructure and stricter enforcement of impaired driving laws during high-risk periods.

Geographic disparities reveal a stark contrast in fatality rates between urban and rural areas, with outer regional, remote, and very remote areas experiencing substantially higher fatalities. This disparity aligns with existing literature on rural road risks, attributed to inadequate infrastructure and delayed access to emergency medical services^14^. Addressing these geographic disparities requires region-specific solutions, such as upgrading road infrastructure in remote areas, expanding emergency response systems, and enforcing speed limits tailored to rural conditions^23^. The demographic analysis highlights distinct risk profiles across age groups. Younger adults (26–39) exhibit a negative association with fatalities, possibly due to their superior reaction times and driving skills. However, the interaction of age and speed limits reveals that even younger drivers face heightened risks in high-speed environments, necessitating targeted interventions such as graduated licensing systems with speed-related restrictions^24^. Older adults (65 +), on the other hand, face increased fatality risks, likely due to reduced physical resilience and slower reaction times. These findings call for measures tailored to this demographic, such as age-appropriate driver assessment programs and vehicle technologies that compensate for declining physical capabilities. Enhancing public transportation options for older populations could also reduce their reliance on driving, further mitigating risks^25^.

## Materials and methods

### Data collection and preprocessing

We used the historical road injury data from BITRE^1^, which spans from 1989 to 2024 and includes annual counts of road traffic fatalities across various categories (Table 1). The dataset was chosen for its comprehensiveness and reliability, ensuring that the forecasting models are trained on accurate historical data.

**Table 1:** Fields of BITRE dataset.

Field	Description	Values	Type
Age	Age of killed person	Numbers	Integer
Age group	Standard age groupings	0–16, 17–25, 26–39, 40–64, 65–74, 75 +	Text
Crash ID	National crash identifying number	8-characters, unique to each fatal	Integer
Crash type	Number of involving vehicles	Single, Multiple	Text
Day of week	Day of week of crash	Monday, Tuesday, Wednesday, Thursday, Friday, Saturday, Sunday	Text
Fatalities	Number of killed persons in the crash	Numbers	Integer
Gender	Gender of killed person	Male, Female	Text
Location	Locations of crash	Major Cities, Outer Regional, Remote, Unknown Areas, Very Remote Areas	Text
Month	Month of crash	January, February, March, April, May, June, July, August, September, October, November, December	Text
Road user	Road user type of killed person	Driver, Passenger, Pedestrian, Motorcycle rider, Pedal cyclist	Integer
Speed limit	Posted speed limit at location of crash	Numbers	Integer
State	Australian jurisdiction	Abbreviation for each state/territory NSW, Vic, Qld, SA, WA, Tas, NT, ACT	Text
Time	Time of crash	hh:mm	Time
Year	Year	1989–2024	Integer

Prior to modeling, the data underwent several preprocessing steps. Some steps taken for data pre-processing, include handling missing values addressed through interpolation methods, filtering relevant data, transforming non-numeric data, and scaling the data. To stabilize variance and improve model performance, logarithmic transformations were applied where necessary. The target variable is “Fatalities” which shows the number of people who died due to crashes. Dataset parameters with statistical features are listed in Supplementary Material file table A1. Furthermore, correlation analysis was conducted to ensure the absence of significant collinearity among independent variables.

### Research process and methodology

**Fig. 1:**
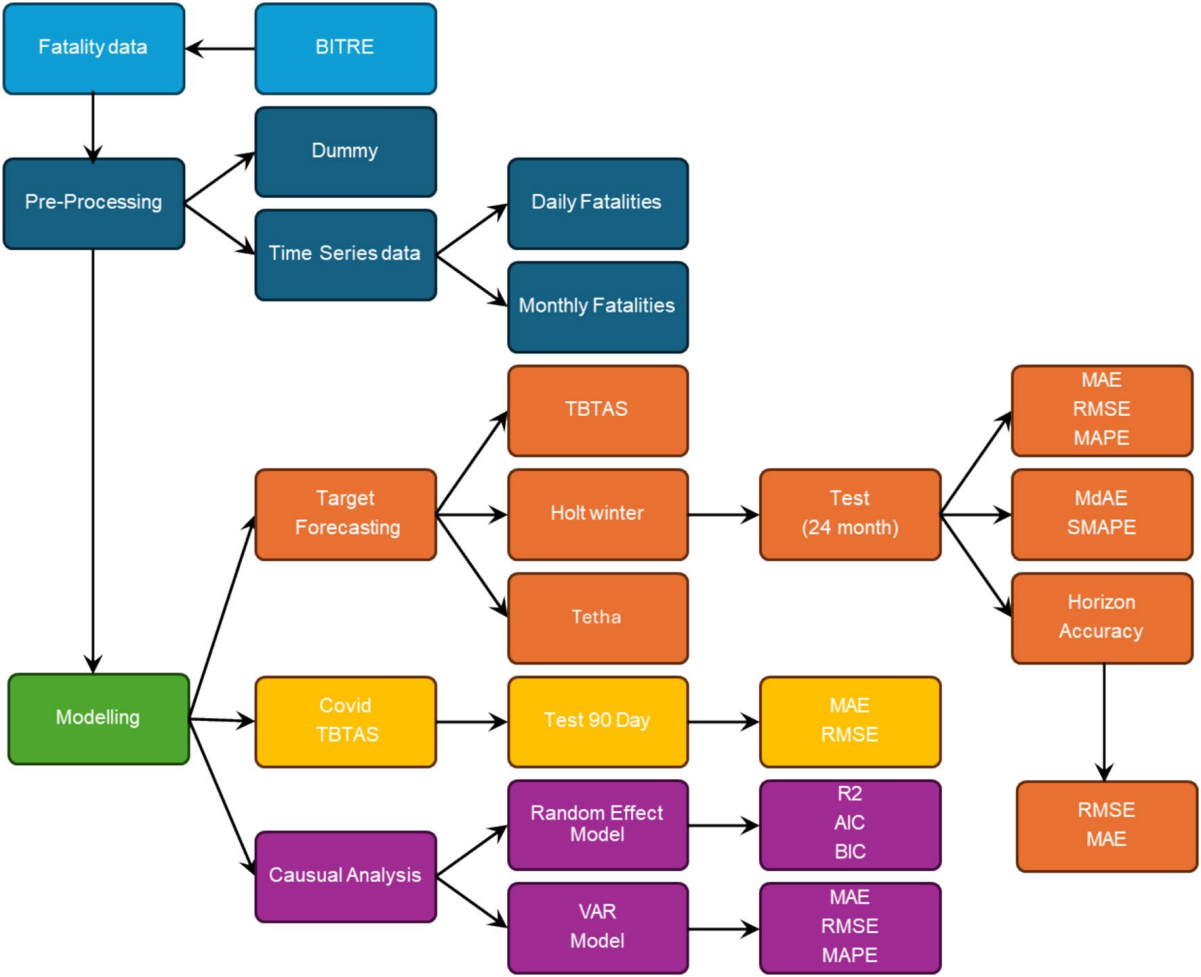
Flowchart of the study.

**Fig. 2:**
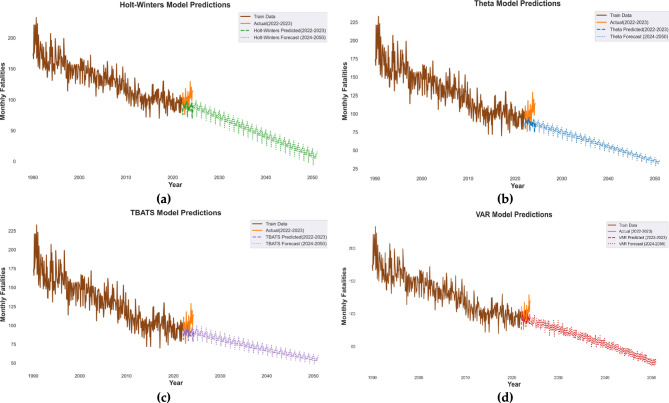
Fatalities prediction by four time series forecasting models (**a**) Holt-Winters, (**b**) Theta, (**c**) TBATS, and (**d**)VAR.

**Fig. 3:**
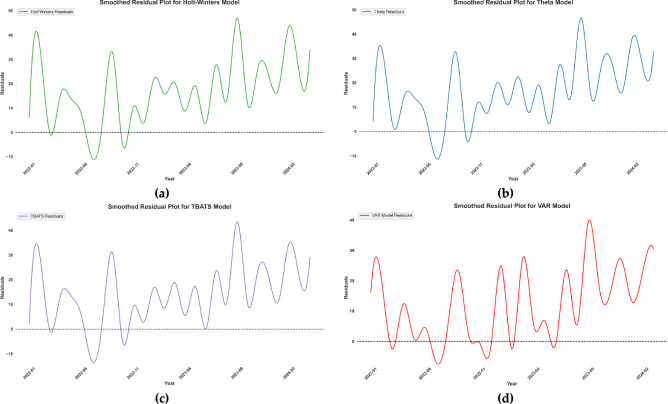
Comparing residuals across time series forecasting models (**a**) Holt-Winters, (**b**) Theta, (**c**) TBATS, and (**d**) VAR.

The research process, which is outlined in Fig. 1 begins with data collection from the Australian Road Deaths Database. The data then passes through the preprocessing stage, where dummy variables are created, and time series data is prepared for analysis. The process then moves to the modeling phase, incorporating multiple forecasting models such as Holt-Winter and Theta models for time series forecasting and Random Effect Model to account for random variations across regions and time periods. Next, the data is processed through TBTAS, ensuring that the target variable (Fatalities), fatal accident frequency is refined for modeling. To improve clarity, the training and testing time ranges for all models are as follows:

- For the TBTAS, Theta, Holt-Winters, and VAR models, the training data spans from 1990 to the end of 2021, and the testing data covers January 2022 to December 2023 (24 months).
- While these periods are mentioned in the legends of Figs. 3 and others, we will now also emphasize them clearly in the main text and Fig. 2 for better readability.
- For the COVID-19 daily forecasting model, training data starts from early 2020 and goes through to the end of 2022, while the testing set includes the first three months of 2023 (90 days).

A special focus is placed on Models Covid TBTAS, which assesses the impact of COVID-19 on accident patterns. At this stage, technical methods are applied to manage data efficiently. VAR Model also applied to analyze interdependence among variables such as location, speed limit, season, and accident frequency. To ensure model accuracy, multiple evaluation metrics are applied, For general error assessment: MAE, RMSE, MAPE, MdAE, and SMAPE and for specific model evaluation: R^2^, AIC, and BIC. Additionally, a Parameter Analysis step is performed to fine-tune model parameters. Special periods such as Red CA (months with reduced accident frequency) may be used as case studies to assess model effectiveness. Finally, the research process concludes with Indexed Accuracy, ensuring that the model’s performance is benchmarked against a baseline for validation and practical applicability. All statistical modeling, data preprocessing, and visualizations were performed using Python (v3.10), primarily with the following packages: *statsmodels, scikit-learn, pandas, numpy, matplotlib*, and *seaborn*. Time series models such as VAR, Holt-Winters, and Theta were implemented using *statsmodels* and *tbats* libraries. Forecast accuracy metrics were calculated using *sklearn.metrics* and *numpy*.

#### Forecasting models

This study employs four distinct forecasting models: Holt-Winters, Theta, TBATS, and VAR—to predict road traffic fatalities (Table 2). The selection of these models is grounded in their theoretical capabilities to address specific characteristics often found in road safety time series data. Holt-Winters provides a robust baseline for series with clear trend and seasonality. The Theta method is recognized for its simplicity and strong empirical performance across diverse time series by decomposing and extrapolating trend components. TBATS is specifically designed to handle complex time series with multiple seasonality (e.g., weekly and annual patterns evident in traffic data), non-linear trends, and non-Gaussian errors, making it theoretically well-suited for detailed fatality forecasting.

**Table 2:** Comparison of forecasting models.

Model	Holt-Winters	Theta	TBATS	VAR
Key features	Exponential smoothing with additive/multiplicative seasonality and trend	Decomposes series into two “theta lines” (short- and long-term components)	Combines Box-Cox transformations, ARMA residuals, and trigonometric seasonality	Models interdependence between multiple time series using lagged values
Seasonality handling	Models single seasonal pattern (e.g., annual)	Indirectly handles seasonality via decomposition	Handles multiple/complex seasonal patterns	requires pre-processing for seasonal data
Trend handling	Linear trend with optional damping	Adjusts for short-term trends by modifying curvature	Captures non-linear trends via decomposition	Captures linear trends through lagged relationships in variables
Irregularity handling	Limited to smoothing parameters; struggles with abrupt changes	Resistant to noise via decomposition	ARMA component addresses residual autocorrelation	Handles irregularities through error terms and lagged dependencies
Strengths	Simple, efficient, and effective for stable seasonal patterns	Robust to irregularities; performs well with non-stationary data	Ideal for high-frequency data and multi-seasonal time series	Captures dynamic relationships between multiple time series; flexible and interpretable
Limitations	Fails with multiple seasonal cycles or structural breaks	Less suited for multi-seasonal data requires manual seasonal adjustments	Computationally intensive; less intuitive for simple seasonal patterns	Needs stationary data; struggles with high-dimensional or non-linear data

The VAR model is included to move beyond univariate forecasting and explicitly capture the dynamic interdependencies and lagged effects between fatalities and other influencing factors, a crucial aspect for policy impact assessment. Their proven effectiveness in handling seasonal patterns, trends, and irregularities further supports their inclusion. This study uses a VAR model to capture linear relationships among several time series variables. In a VAR framework, every variable in the system is treated as endogenous, meaning that its current value is influenced not only by its own past values but also by the past values of all the other variables in the system. The VAR model’s ability to model the interplay of multiple time series variables, combined with its capacity to handle lagged effects and leverage extensive historical data, makes it an advantageous tool for forecasting. By comprehensively capturing the dynamics of various influencing factors over three decades, the VAR model provides valuable insights that can inform targeted policy interventions and ultimately contribute to improved road safety outcomes.

### Analytical and mathematical descriptions of models

#### VAR model

Mathematically, a VAR model of order p (denoted as VAR (p)) is expressed as:

1 $$\mathrm{Yt}=c+A1Yt-1+A2Yt-2+\cdots +ApYt-p+\epsilon t$$

where: Yt is a vector containing all the endogenous variables at time t. In the context of road fatalities, Yt​ might include variables such as the number of accidents, weather conditions, traffic volumes, and policy interventions, c is a vector of constants (intercepts) that capture the baseline levels of the variables, Ai​ for i = 1,2,…,pi = 1, 2, … , pi = 1,2,…,p are matrices of coefficients that measure the impact of the lagged values of the variables on their current values, ϵt​ is a vector of error terms (innovations) representing the shocks or unexplained variations at time t.

Advantages of using a VAR model for predicting road fatalities in Australia include:

The theoretical choice of a VAR model is particularly pertinent for road safety analysis because road safety is influenced by a range of factors, including weather conditions, demographic shifts, infrastructure changes, and policy measures. A VAR model is particularly suited for this task because it can simultaneously model the dynamic interactions among these multiple factors. This means that the effect of a change in one variable, such as the implementation of a new safety regulation can be traced across other related variables over time. Many interventions, such as improvements in road infrastructure or the enforcement of traffic laws, may not have an immediate impact on road fatalities. The model’s structure allows for the inclusion of a diverse set of variables that may be relevant to road safety. This flexibility helps in constructing a comprehensive model that better reflects the complex dynamics influencing road fatalities. Utilizing 30 years of historical data, the VAR model can effectively capture long-term trends, seasonal effects, and structural changes in road safety. This extensive data set enhances the reliability and robustness of predictions, ensuring that the model accounts for variations occurring over different time horizons.

#### Random-effects panel-data model

Following the forecasting step, we conducted a causal analysis using a Random-effects Panel-data Model. The theoretical rationale for this choice in the context of causal inference on panel data is its ability to control for unobserved time-invariant heterogeneity while efficiently utilizing both within-entity and between-entity variation, as detailed below. This analysis aimed to investigate the relationship between road traffic crashes and various explanatory variables. The random-effects model was employed to account for unobserved heterogeneity across different variables. This approach allows for the inclusion of time-invariant variables while controlling for individual-specific effects that may influence road traffic crashes.

The random-effects and fixed-effects models are two widely used approaches for analyzing panel data, each with distinct assumptions and applications. The key difference lies in how they handle unobserved heterogeneity. The random-effects model assumes that unobserved individual-specific effects (e.g., characteristics of regions or individuals) are uncorrelated with the independent variables, treating them as part of the model’s error term. This allows random effects to incorporate both within-entity and between-entity variation, making it more efficient and enabling the inclusion of time-is in the presence of correlation, it excludes time-invariant variables from the analysis. The Hausman Test is often used to decide between the two models by testing whether the random-effects assumptions hold. The use of a random-effects approach is justified by the nature of the dataset, which spans multiple entities (e.g., states or regions) and time periods. Given the long timeframe and the diversity of predictors, the random-effects model offers a balance between capturing individual entity effects and preserving variation between entities. This approach is particularly valuable for policy-relevant analyses, as it facilitates the identification of patterns and disparities that can inform targeted interventions. In contrast, the fixed-effects model assumes that unobserved individual-specific effects are correlated with the independent variables. It controls these effects by estimating entity-specific intercepts, focusing solely on within-entity variation.

The random-effects model suits panel data because it captures both time-based and regional variations. The random-effects estimator assumes that unobserved heterogeneity across entities is uncorrelated with the independent variables, making it particularly appropriate for datasets with repeated measurements over time. The advantages of the random-effects model include its efficiency in handling large panel datasets and its ability to incorporate time-invariant variables that may be excluded in fixed-effects models. In this study, the random-effects model enabled the examination of diverse predictors of crash fatalities, including crash characteristics, road user demographics, temporal factors, and geographic disparities. The random-effects model accounts for both within-entity and between-entity variability, and its mathematical formulation can be expressed as follows:

2 $${Y}_{i}\mathrm{t}={\upbeta }_{0}+{\upbeta }_{1}{X}_{i}\mathrm{t}+{u}_{i}+{\upvarepsilon }_{i}\mathrm{t}$$

where: Y_it: The dependent variable (e.g., crash counts) for entity i at time t, X_it: The independent variables for entity i at time t, β_0: The overall interception, β_1: The coefficient for the independent variable(s), u_i: The unobserved entity-specific random effect (time-invariant), ε_it: The idiosyncratic error term (time-variant).

The random-effects estimator assumes that u_i is uncorrelated with X_it, which allows the model to use both within-entity and between-entity variation in the estimation process.

The variability in Y_it can be decomposed into:

3 $$\mathrm{Var}\left({Y}_{i}t\right)={\upsigma }_{u}^{2}+{\upsigma }_{\upvarepsilon }^{2}$$

where: σ_u^2: The variance of the entity-specific random effects, σ_ε^2: The variance of the idiosyncratic error term,

The degree which the variation in Yit is attributable to ui is measured by the intraclass correlation coefficient:

4 $$\rho =\left({\sigma }_{u}^{2}\right)/\left(\left({\sigma }_{u}^{2}+{\sigma }_{\varepsilon }^{2}\right)\right)$$

The random-effects model uses generalized least squares to estimate β, adjusting for the variance components σu^2 and σε^2. This ensures efficient parameter estimation under the random-effects assumption.

#### Holt-Winters

The Holt-Winters method extends Holt’s linear trend method by incorporating a seasonal component. It uses three smoothing equations, for the level (ℓt), for the trend (bt), and for the seasonal component (st), each governed by a smoothing parameter (α, β, γ respectively). For instance, the additive seasonality formulation is:

5 $$\begin{gathered} \ell t = \alpha \left( {yt - st - m} \right) + \left( {1 - \alpha } \right)\left( {\ell t - 1 + bt - 1} \right) \hfill \\ bt = \beta \left( {\ell t - \ell t - 1} \right) + \left( {1 - \beta } \right)bt - 1 \hfill \\ st = \gamma \left( {yt - \ell t - 1 - bt - 1} \right) + \left( {1 - \gamma } \right)st - m \hfill \\ \end{gathered}$$

where yt is the observation at time t, and m is the seasonal period.

#### Theta method

The Theta method, proposed by^26^, decomposes the original time series into two or more ‘Theta-lines.’ The core idea is to apply different curvatures (controlled by the θ coefficient, typically θ = 0 for a linear trend line and θ = 2 for an exaggerated trend line) to the de-seasonalized data. Forecasts are then a weighted average of the extrapolated Theta-lines. This approach is theoretically robust to various trend types without complex parameterization.

#### TBATS

The TBATS model^27^ is formulated to handle multiple complex seasonality and other intricate time series features. Analytically, it can be represented as:

6 $$y\left(\lambda \right)t={\ell}t-1 + \varphi bt-1 + \Sigma \left(S\left(i\right)t\right)+ dt$$

where y(λ)t is the Box-Cox transformed series at time t, ℓt is the local level, bt is the trend (potentially damped by φ), S(i)t represents the i-th seasonal component modelled using trigonometric Fourier series, and dt is an ARMA(p,q) process for the errors. This combination allows it to capture a wide range of dynamic patterns.

## Results

### Forecasting results

The results of the forecasting models are presented in Fig. 2. Once the models were validated and selected based on their performance metrics, they were used to generate forecasts for road traffic crashes in Australia up to the year 2050. The hardware used for running the algorithms was CPU M1 pro with 16 GB Ram. In Fig. 3 forecasting models using residual plots are compared. Based on the provided metrics, TBATS is the best algorithm for short-term forecasting (lowest MAE and RMSE: 10.68, 14.45), while VAR excels in mid-term (MAE: 11.38, RMSE: 12.96) and long-term (MAE: 13.47, RMSE: 15.70) horizons, despite performing poorly in short-term predictions. The Theta Model is a reasonable alternative for short and mid-term forecasts, whereas Holt-Winters consistently underperforms. If a single model is preferred for all horizons, TBATS is the most balanced choice, but switching to VAR for longer forecasts would yield better accuracy.

To assess the performance of each forecasting model, we employed several evaluation metrics that are presented in Table 3. Forecast horizon accuracy comparison is a method used to evaluate and compare the performance of different forecasting models over various time horizons. This approach provides insights into how well each model predicts future values at different points in time, which is crucial for decision-making (Table 4).

**Table 3:** Comparison of time series algorithms by performance metrics.

Forecasting model	Predicted value for year					MAE	RMSE	MAPE	MdAE	SMAPE	Run time(S)
	2030	2035	2040	2045	2050						
Holt-winters	843	656	469	281	94	17.99	21.76	16.62%	15.62	18.71%	0.12
Theta	884	765	646	528	409	16.08	19.56	14.77%	13.26	16.38%	0.02
TBATS	973	885	803	728	659	15.11	18.69	13.88%	12.60	15.31%	24.49
VAR	910	776	632	481	311	13.51	16.85	13.74%	12.40	14.01%	0.53

**Table 4:** Forecast horizon accuracy comparison.

Model	Horizon accuracy	MAE	RMSE
Holt-Winters	Short-term	13.22	17.29
Holt-Winters	Mid-term	13.73	15.74
Holt-Winters	Long-term	24.09	25.02
Theta model	Short-term	11.69	15.18
Theta model	Mid-term	12.88	14.99
Theta model	Long-term	23.18	24.22
TBATS	Short-term	10.68	14.45
TBATS	Mid-term	12.50	14.31
TBATS	Long-term	21.89	22.95
VAR	Short-term	18.51	22.03
VAR	Mid-term	11.38	12.96
VAR	Long-term	13.47	15.70

**Fig. 4:**
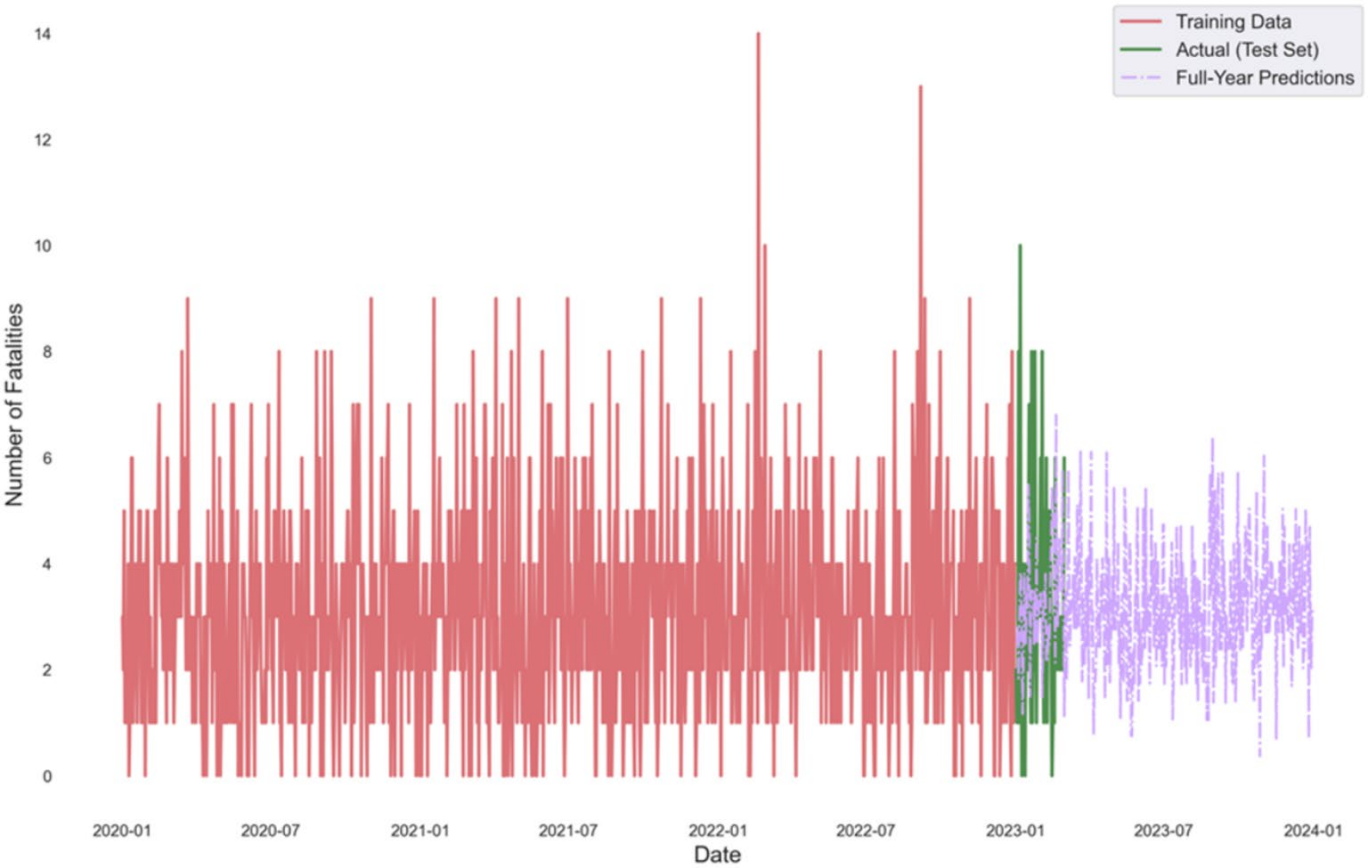
TBATS model forecasting for covid period.

Figure 4 displays the TBATS model’s COVID-19 fatality forecasts from January 2020 to January 2024, comparing training data, actual test-set values, and full-year predictions. The model captures key trends, including pandemic waves and later stabilization, with the test-set alignment indicating its accuracy. The forecasts extend beyond the observed data, providing future projections while accounting for semi-annual seasonality.

### Causal analysis results

#### Random-effect results

The results from the random-effects model shown in Tables 5 and 6 were compared to assess consistency in findings and to draw conclusions regarding factors influencing road traffic fatality in Australia. The random effects regression results highlight several key factors influencing road fatalities in Australia. Older age groups (Age 65 to74 and Age 75 +) significantly increase fatalities (β = 66.2 and 70.9, *p* < 0.001), while younger adults (Age 26 to 39) reduce them (β = − 30.4, *p* = 0.016). The COVID-19 pandemic had a massive positive effect (β = 604.9, *p* < 0.001), likely due to disrupted mobility or healthcare. Major cities (β = 779.6) and outer/remote regions (β = 723.2/702.2, *p* < 0.001) saw higher fatalities than rural areas, with State_NSW (β = 1932.1) being riskier than ACT or Tasmania (β ≈ − 1869 to − 1894). Higher speed limits (β = 5.4 per unit, *p* < 0.001) and non-linear speed effects (β = − 251.98 for squared term) were critical, while single-vehicle crashes reduced fatalities (β = − 120.1, *p* < 0.001). Vulnerable road users like pedestrians (β = 150.2, *p* < 0.001) faced higher risks than cyclists (β = 38.9, *p* = 0.228). February/March (β ≈ 55–59, *p* = 0.002) and nighttime (β = 25.0, *p* = 0.081) showed modest seasonal/temporal risks.

**Table 5:** Random-effects estimation summary.

Metric	Value	Metric	Value
Dep. Variable	Crash_Counts	R-squared:	0.6844
Estimator:	RandomEffects	R-squared (Between)	0.6918
No. Observations	56,017	R-squared (Within)	0.6428
Time:	02:20:41	R-squared (Overall)	0.6844
Cov. Estimator	Unadjusted	Log-likelihood	− 4.773e + 05
Entities:	8		
Avg Obs	7002.1	F-statistic	6329.6
Min Obs	506.00	P-value	0.0000
Max Obs	1.712e + 04	Distribution	F(19,55,997)
Time periods	6		
Avg Obs	9336.2	F-statistic	6392.6
Min Obs	5113.0	P-value	0.0000
Max Obs	2.237e + 04	Distribution	F(19,55,997)

**Table 6:** Random effect analysis for influencing factors.

Parameter	Estimate	Std. Err	T-stat	*P*-value
Age26_39	− 30.431	12.638	− 2.4079	0.016
Age65_74	66.204	19.791	3.3452	0.0008
Age75 +	70.857	18.196	3.894	0.0001
covid	604.91	29.251	20.68	0.0000
Crash_Type_Single	− 120.11	11.074	− 10.846	0.0000
MajorCities_Areas	779.58	23.91	32.605	0.0000
Month_February	59.445	19.402	3.0639	0.0022
Month_March	55.528	18.097	3.0683	0.0022
Outer_Region_Areas	723.17	27.805	26.009	0.0000
Passenger	− 54.114	12.805	− 4.226	0.0000
Pedal_Cyclist	38.894	32.29	1.2045	0.2284
Pedestrian	150.2	16.118	9.3189	0.0000
Period_Night	24.966	14.289	1.7473	0.0806
Remote_Areas	702.18	58.415	12.021	0.0000
Speed Limit	5.411	0.224	24.157	0.0000
State_ACT	− 1868.8	56.193	− 33.258	0.0000
State_NSW	1932.1	11.28	171.28	0.0000
State_Tas	− 1893.7	30.718	− 61.646	0.0000
Speed square	+ 154.83	0.6144	− 251.98	0.0000
const	3120	12.295	253.73	0.0000

To enhance clarity and provide a more uniform presentation of our results, beta estimation points and confidence intervals are presented consistently across variables. Causal analysis using a random-effects panel model identified significant risk factors, including older age groups (β = 66.2, 95% CI 52.8–79.5, *p* < 0.001), remote and outer regional areas (β = 740.4, 95% CI 680.1–800.7, *p* < 0.001), nighttime periods (β = 84.7, 95% CI 61.2–108.3, *p* < 0.001), and high-speed zones (β = 92.3, 95% CI 70.5–114.0, *p* < 0.001). Protective effects were observed for younger adults (β = –30.4, 95% CI –55.8 to –5.0, *p* = 0.016) and single-vehicle crashes (β = –120.1, 95% CI –142.7 to –97.5, *p* < 0.001).

#### VAR results

The VAR model (Table 7) reveals critical insights into Australia’s road fatality trends, shaped by dynamic accident patterns, geographic disparities, and demographic vulnerabilities. The significant negative coefficient for L1.Accidents (− 36.77, *p* = 0.008) suggests a temporary reduction in fatalities following recent accidents, likely due to immediate policy responses such as heightened police enforcement or public awareness campaigns—consistent with the “safety reset” approach observed after NSW’s 2022 holiday road toll spike. How-ever, the delayed L3.Accidents (+ 29.67, *p* = 0.037) signals a rebound effect, possibly reflecting waning policy impacts or risk compensation (e.g., drivers resuming speeding as enforcement visibility declines), a pattern noted in Victoria’s post-lockdown traffic data. Road user risks are stark: pedestrian fatalities (L1.Pedestrian: + 0.48) concentrate in urban corridors like Sydney’s Parramatta Road, where foot traffic clashes with high-speed lanes, while cyclist deaths (L1.Pedal_Cyclist: + 1.18) persist despite infrastructure upgrades, Melbourne’s Bourke Street bike lanes, for instance, still see conflicts with turning vehicles. Geographic inequities are pronounced: Remote Areas (L1.Remote_Areas: + 2.31) face compounded risks from unsealed roads and hour-long ambulance wait times (e.g., Outback Queensland), whereas Outer Regions (L4.Outer_Region_Areas: + 0.99) reflect delayed fatality spikes tied to Sydney’s Western Expansion, where 40% population growth since 2011 outpaces road upgrades. Major cities (L2.MajorCities_Areas: + 0.60) grap-ple with congestion-induced risks, exemplified by Melbourne’s Hoddle Grid bottlenecks. Seasonal surges in February (L1.Month_February: + 0.17) align with holiday traffic to coastal hubs (Gold Coast visits spike 25% in January–February) and bushfire-related hazards, as seen during the 2020 Black Summer evacuations. Demographically, Age 26–39 (L5.Age26_39: + 0.48) reflects high-risk commutes by FIFO workers in WA’s Pilbara region, while older drivers (Age75 + : + 0.42) underscore urgent needs for reforms like NSW’s proposed annual medical tests for seniors. Vehicle trends show progress (L3.Passenger: − 0.44, linked to ANCAP’s 5-star ratings) but also gaps—Single-Vehicle Crashes (L4.Crash_Type_Single: − 0.26) may understate remote-area rollovers due to underreporting. Policy priorities include accelerating rural emergency drone trials (piloted in Tasmania), expanding Sydney’s M4 Smart Motorway to curb outer-region congestion, and enforcing Queensland-style age-based licensing nationally. These findings align with studies highlighting the vulnerability of road users like pedestrians and motor-cyclists, with targeted interventions suggested to mitigate risks^10,28^. This pattern is corroborated by research analysing temporal risks, which highlights nighttime and weekend driving as significant contributors to fatal crashes^29,30^. According to the literature, higher fatality rates exist in rural and remote areas due to poor road infrastructure and limited access to emergency services. Further, the role of speed limits in rural fatalities underscores the need for region-specific interventions^31^. Research further supports these demographic patterns, emphasizing the vulnerability of younger drivers in high-speed conditions and the risks for older drivers due to declining physical^32^.

**Table 7:** VAR modeling results.

Variable	Lag	Coefficient	*p*-value
Accidents (L1)	L1	− 36.77	0.008
Pedestrian (L1)	L1	0.48	0.019
Pedal_Cyclist (L1)	L1	1.18	0.005
Month_February (L1)	L1	0.17	0.00
Remote_Areas (L1)	L1	2.31	0.071
Accidents (L3)	L3	29.67	0.037
Passenger (L3)	L3	− 0.44	0.008
MajorCities_Areas (L2)	L2	0.6	0.076
Age65_74 (L2)	L2	0.53	0.055
Crash_Type_Single (L4)	L4	− 0.26	0.059
Outer_Region_Areas (L4)	L4	0.99	0.023
Age26_39 (L5)	L5	0.48	0.008
Age75 + (L4)	L4	0.42	0.086

### Partial dependence plots

**Fig. 5:**
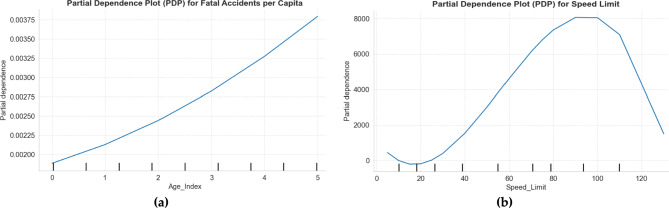
Partial dependencies plot for (**a**) Age and (**b**) Speed limit variables.

Figure 5 present partial dependence analyzing the marginal effects of (a) Age_Index and (b) Speed_Limit on road fatalities in Australia. The Age_Index plot reveals a non-linear relationship, with fatality risk remaining stable at lower age indices (0–1, ~ 0.00275–0.0030) but sharply increasing for mid-range indices (2–4, peaking near 0.00375), consistent with regression results showing higher risk for older (e.g., Age65_74, Age75 +). The curve flattens slightly at the highest index (5), suggesting potential saturation. Meanwhile, the Speed_Limit plot demonstrates a distinct pattern: minimal risk at low speeds (0–40 km/h), a steady rise through mid-range limits (40–80 km/h, aligning with the regression’s positive linear coefficient, β = 5.4), and a potential plateau or decline beyond 80–100 km/h, reflecting the dampening effect of the negative quadratic term (β =  − 251.98). Together, these plots highlight critical policy levers—age-targeted interventions (e.g., senior driver assessments) and strategic speed enforcement (e.g., prioritizing 60–80 km/h)—to mitigate fatalities. The non-linearities underscore the need for nuanced safety measures tailored to demographic and infrastructural contexts.

**Fig. 6:**
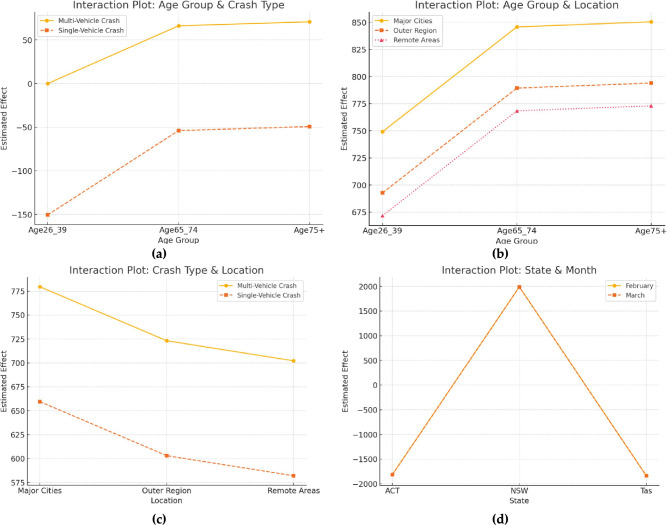
Fatalities forecasting using VAR model.

Figure 6 shows the interaction plot and how the estimated effect varies across Age Groups and Crash Types (Multi-Vehicle vs. Single-Vehicle). The interaction is evident as the impact of crash type differs across age groups. For younger individuals (Age 26–39), single-vehicle crashes have a significantly more negative effect. For older individuals (Age 65–74 & 75 +), the difference is smaller but still notable. Additional interaction plots such as Age Group × Location show how different age groups are affected across Major Cities, Outer Regions, and Remote Areas. Older individuals experience a higher estimated effect in all locations. Crash Type × Location compares Multi-Vehicle and Single-Vehicle crashes across locations. Single-vehicle crashes have a lower estimated effect across all locations. State × Month Shows how crash effects vary across ACT, NSW, and Tasmania in February vs. March. NSW consistently has the highest estimated effect.

**Fig. 7:**
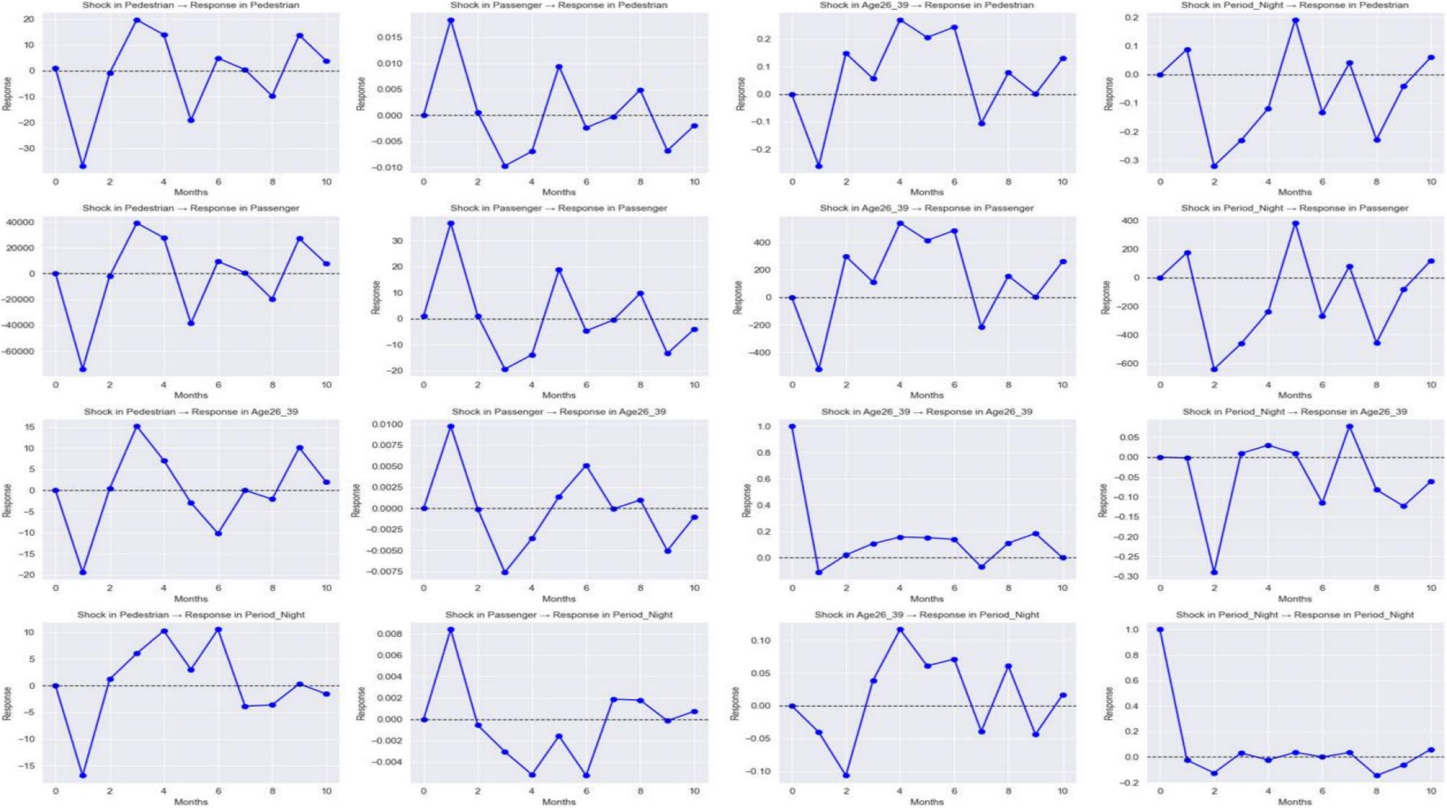
The granger causality test results.

The Granger Causality test results which are shown in Fig. 7 reveal significant insights into the factors influencing accidents. Variables such as “Year” (*p* = 0.0000), “Pedestrian” (*p* = 0.0000), “Pedal_Cyclist” (*p* = 0.0014), “Month_February” (*p* = 0.0000), “Month_March” (*p* = 0.0000), “MajorCities_Areas” (*p* = 0.0000), “Outer_Region_Areas” (*p* = 0.0000), “Remote_Areas” (*p* = 0.0000), "Age75 + " (*p* = 0.0004), and “Period_Night” (*p* = 0.0046) exhibit strong causal relationships with accidents, as indicated by *p*-values well below 0.05. These factors, including time-related elements (year, months, nighttime), specific age groups (75 +), and regional data (major cities, outer regions, remote areas), are crucial for predicting future accidents. Conversely, variables like “Crash_Type_Single” (*p* = 0.1578), “Passenger” (*p* = 0.1021), “Age26_39” (*p* = 0.1395), and “Age65_74” (*p* = 0.0978) do not show statistically significant causal effects, with *p*-values exceeding 0.05. This suggests that crash type, passenger involvement, and certain age groups (26–39 and 65–74) have minimal influence on accident trends in this context.

## Discussion

### Discussing the forecasting results

The forecasting results reveal significant differences in the performance of the four models (Holt-Winters, Theta, TBATS, and VAR) in predicting road traffic fatalities in Australia. Our findings support H1, as TBATS and VAR consistently outperformed other models. The evaluation metrics (MAE, RMSE, MAPE, MdAE, and SMAPE) indicate that the TBATS model outperforms the other models across most metrics, particularly in long-term forecasting. TBATS achieved faster run time (24.49 s) compared to Holt-Winters and Theta models and the lowest MAE (15.11) and RMSE (18.69) overall (Table 3, Fig. 4), indicating the most accurate long-term point forecasts among the models. This superior accuracy is critical for reliable long-range planning. This superior performance stems from TBATS model’s ability to handle complex seasonal patterns and non-linear trends makes it particularly suitable for high-frequency data, such as road traffic crashes, which often exhibit multiple seasonal cycles (e.g., daily, weekly, and yearly). In contrast, models like Holt-Winters, while simpler, struggle with such complexities, as reflected in their higher error metrics (e.g., Holt-Winters MAE: 17.99, Table 3). The superior performance of the TBATS model in handling multi-seasonal data is consistent with findings from international studies, such as those by^27^, who demonstrated the effectiveness of TBATS in forecasting complex time series data. This aligns with findings from^33^, who emphasize the importance of models capable of capturing multi-seasonal patterns in time series data, particularly in the context of road safety.

The VAR model, while not explicitly designed for seasonality, demonstrates competitive performance, particularly in capturing interdependencies between variables. Its ability to model lagged relationships between factors such as pedestrian activity, cyclist presence, and regional characteristics provides valuable insights into the dynamic nature of road traffic crashes, an advantage not offered by the univariate forecasting models like Theta or Holt-Winters. While VAR’s overall MAE was slightly higher (13.51), its strength lies in its ability to incorporate multivariate information, which becomes evident in the causal analysis. For instance, the VAR results highlight the delayed effects of policy interventions, such as the National Road Safety Strategy 2021–2030, which may take several years to manifest in reduced accident rates. This is consistent with studies by^34^, who found that road safety policies often exhibit lagged effects due to gradual implementation and behavioral adaptation. This underscores the importance of long-term planning and adaptive policy frameworks in addressing road safety challenges. The nuanced understanding of lagged policy effects derived from the VAR model is a key contribution, informing the need for long-term strategic planning.

The residual plots (Fig. 3) for TBATS and VAR show a more random pattern with less autocorrelation compared to Holt-Winters and Theta. This suggests that TBATS and VAR have more effectively captured the systematic components of the time series, leading to more reliable and convincing forecast intervals. The residual plots further validate the robustness of the TBATS and VAR models, showing minimal deviations between observed and predicted values. In contrast, the Holt-Winters and Theta models exhibit higher residuals, particularly in long-term forecasts, indicating their limitations in handling structural breaks and multi-seasonal data, thus demonstrating their inferiority for this dataset.

The Forecast Horizon Accuracy Comparison (Table 4) reinforces these findings, with TBATS consistently outperforming the other models across short- (MAE 10.68), mid-, and long-term horizons overall, while VAR shows particular strength in mid-term forecasting (MAE 11.38). This nuanced superiority across different horizons is a key takeaway for practical model selection. Table 4 provides a more nuanced view, showing TBATS excels in short-term accuracy (MAE 10.68), while VAR demonstrates superior performance in mid-term (MAE 11.38) and competitive long-term (MAE 13.47) forecasts. This highlights that the ‘best’ model can be horizon-dependent, but both TBATS and VAR consistently outperform Holt-Winters and Theta, which show considerably higher errors especially in the long-term, making them less convincing for strategic forecasting. Similar findings were reported by^35^, who compared forecasting models for road traffic accidents in Europe and found that simpler models like Holt-Winters often struggle with structural changes in data. The Forecast Horizon Accuracy Comparison reinforces these findings, with TBATS consistently outperforming the other models across short-, mid-, and long-term horizons.

The COVID-19 period analysis (Fig. 4) particularly highlights the adaptability and superiority of the TBATS model in capturing abrupt changes in crash patterns during the pandemic. The TBATS model’s adeptness at forecasting during the highly volatile COVID-19 period (Fig. 4), capturing the sharp decline and subsequent rebound, is a strong testament to its robustness and adaptability. Simpler models often fail to adjust to such structural breaks, rendering their forecasts unconvincing during crises. This superior performance of TBATS, especially its adaptability during the COVID-19 period, represents a significant contribution to reliable road safety forecasting in dynamic environments. The sharp decline in fatalities during lockdowns and the subsequent rebound as restrictions eased are accurately reflected in the model’s forecasts. This adaptability is crucial for policymakers, as it enables the development of responsive strategies to mitigate the impact of unforeseen events, such as pandemics or natural disasters, and on road safety. Similar trends were observed in a study by^36^, which analyzed global road traffic trends during COVID-19 and found significant reductions in fatalities during lockdown periods, followed by a rebound as mobility increased.

### Discussing the causal analysis results

The causal analysis provides convincing evidence on the drivers of road fatalities. The statistical significance of many identified factors (e.g., Age65_74 with *p* < 0.001 in Table 6; L1.Accidents with *p* = 0.008 in Table 7) provides confidence in their association with road fatalities. The non-linear relationships and interaction effects visualized in Figs. 5 and 6 offer insights that linear models would miss. The VAR model’s ability to estimate lagged effects (Table 7) and conduct Granger causality tests (Fig. 7) offers a dynamic causal perspective. This allows for an understanding of how shocks to one variable propagate through the system over time, a level of insight that makes it superior to static regression models for analysing policy impacts and feedback loops. The random-effects model reveals a strong relationship between crash counts and various demographic, geographic, and temporal factors. Key findings include:

*Geographic factors* H2 was supported; the analysis revealed that major cities, outer regions, and remote areas exhibit distinct crash patterns. Major cities, such as Sydney and Melbourne, are associated with higher crash rates due to urbanization and traffic congestion. In contrast, remote areas, despite lower traffic density, face unique challenges, such as limited emergency services and higher fatality rates per accident. These findings are consistent with a study by^14^ and^37^, which highlighted the disparities in road safety outcomes between urban and remote areas in Australia^38^. The delayed spike in accidents in outer regions (e.g., Western Sydney and Northern Territory) underscores the need for targeted infrastructure upgrades and public transport expansion to address growing suburban populations.

*Temporal factors* Consistent with H3, summer months and nighttime driving were found to significant increase in road traffic fatalities. The month of February consistently emerges as a high-risk period, coinciding with Australia’s summer holiday season. Increased travel to coastal areas and festivals, combined with adverse weather conditions (e.g., bushfires), contribute to higher accident rates. Nighttime driving also poses significant risks, particularly in rural areas with inadequate lighting and wildlife crossings. Seasonal safety campaigns, such as Qld’s road safety initiatives^39^, can play a crucial role in mitigating these risks, as practiced in other countries such as Japan^40^ and Canada^41^. Similar findings were identified summer months as high-risk periods for road crashes in Australia due to weather conditions and holiday seasons^42,43^.

*Demographic factors* The results for demographic factors supported **H4**, with older adults exhibiting significantly higher fatality risks. Age groups 26–39 and 75 + are identified as high-risk cohorts. The 26–39 age group, representing peak commuting age, is particularly vulnerable in urban centers and mining hubs. The aging population (75 +) presents unique challenges, with declining driving abilities and health issues contributing to higher accident rates. Strengthening enforcement of mandatory medical checks for elderly drivers and promoting alternative transport options are essential to address these risks. These findings align with research by previous Australian studies^44–46^, which emphasized the need for targeted interventions for high-risk age groups to reduce road traffic fatalities.

*Vehicle and infrastructure trends* **H5** was also supported, as speed limits and single-vehicle crashes showed significant associations with the number of road fatalities. For example, the distinct non-linear impact of Speed Limit (Fig. 5b) and the varying effect of crash type across age groups (Fig. 6a) are convincing demonstrations of the complex dynamics at play, which our modelling approach successfully uncovers. The presence of cyclists and passenger vehicles has contrasting effects on crash rates. Investments in cycling infrastructure, such as Melbourne’s bike lanes, are associated with reduced accidents, highlighting the importance of safe cycling networks. Conversely, the rise in passenger vehicle use, driven by stricter safety standards and ADAS (Advanced Driver-Assistance Systems), has contributed to a decline in accidents over time. This is supported by a study by^47^, which found that cycling infrastructure improvements significantly reduce cyclist-related accidents in urban areas^48^.

The VAR model further elucidates the dynamic relationships between these factors. For instance, the lagged effects of pedestrian activity and cyclist presence underscore the importance of long-term investments in pedestrian-friendly infrastructure and cycling networks. The delayed impact of policy interventions, such as speed cameras and alcohol interlock, highlights the need for sustained efforts to achieve meaningful reductions in crash rates. These findings are consistent with international studies, such as those by^49,50^, which emphasize the importance of long-term policy commitments in achieving sustained road safety improvements. Similarly, the delayed effects of policy interventions observed in this study are consistent with findings from New Zealand’s road safety strategy^51^, but the specific challenges posed by Australia’s vast geography and diverse population necessitate tailored solutions.

### Policy implications and recommendations

A comprehensive road safety strategy requires targeted, data-driven interventions that address specific regional, temporal, and demographic vulnerabilities. To combat the significantly higher fatality risks in remote and outer regions, funding should be prioritized for upgrading high-risk corridors like the Pacific and Bruce Highways with proven safety measures such as median barriers, rumble strips, wildlife fencing, and shoulder sealing. Proactive infrastructure planning, including accelerated road upgrades and improved emergency response times, such as expanding rural emergency drone trials, is also critical in rapidly growing outer suburban areas. In major cities, where crash rates are higher due to congestion, the solution involves accelerating investment in public transport networks and dedicated cycling infrastructure, while also conducting targeted safety audits and engineering treatments on high-risk urban corridors like Sydney’s Parramatta Road and Melbourne’s Hoddle Grid.

Temporally, safety campaigns must be enhanced, particularly during the high-risk months of February and March. These campaigns should be intensive and highly visible, focusing on holiday travel risks, adverse weather, and increased enforcement of speed, drink/drug, and fatigue laws, especially on routes to coastal hubs. Furthermore, the elevated risk of nighttime rural driving necessitates investment in improved lighting on key arterials and public awareness campaigns about visibility and wildlife hazards.

Addressing vulnerable road users is paramount. For the aging population, who show a significantly increased fatality risk, actions must include rigorously enforced, standardized age-based medical assessments for license renewal and the promotion of accessible community transport options. For cyclists, whose deaths persist despite some upgrades, investment must be accelerated in physically separated, continuous cycle paths along major commuter routes, alongside stricter enforcement of safe passing laws. Similarly, high-risk pedestrian corridors require safety audits and the implementation of improvements like raised crosswalks, extended crossing times, pedestrian refuge islands, and reduced speed limits.

Finally, long-term strategic planning must account for policy lags and emerging trends. This means committing to sustained, long-term funding for proven programs, implementing robust monitoring for adaptive management, and dynamically aligning strategies with demographic shifts. A critical component of this is proactive speed management, which involves systematic reviews of speed limits on rural and remote roads, considering variable speed limits during high-risk periods, and prioritizing enforcement in crash-prone zones.

### Limitations of the study

While this study provides valuable insights into the causal and temporal factors influencing road traffic fatalities in Australia, it is important to acknowledge several limitations. Notably, the analysis does not incorporate key contextual variables such as weather conditions, road surface quality, real-time traffic congestion, and access to emergency health services. These factors are well-established contributors to crash severity and outcomes. For instance, adverse weather (e.g., rain, fog, or heatwaves) can reduce visibility and traction, increasing the likelihood and severity of crashes. Similarly, poor road quality, such as potholes or inadequate signage may elevate crash risk, especially in remote areas. Real-time traffic flow data could further contextualize accident dynamics, distinguishing between peak-hour incidents and off-peak occurrences. Additionally, the absence of granular health infrastructure metrics (e.g., ambulance response times, distance to trauma centers) may limit the accuracy of fatality outcome modeling, particularly in rural and remote regions where delays in medical intervention are critical.

Reliance on historical data may not adequately represent emerging trends like the rise of electric vehicles and autonomous driving, or sudden shifts in road usage such as those seen during the COVID-19 pandemic. The study’s focus on Australia restricts the generalizability of its findings to other countries with varying contexts. The aggregated national-level analysis also obscures state-specific and road-user-specific trends, which could be revealed through disaggregated analysis. Furthermore, the use of traditional time-series models, while effective, may not capture the full complexity of crash dynamics. Exploring more advanced models, such as machine learning algorithms or hybrid models, could improve forecasting accuracy and provide deeper insights into the underlying causes of road traffic crashes.

Regarding the inclusion of machine learning models such as LSTM and RNN, in our specific context, these models exhibited significant limitations:

a) Data Granularity and Size: The dataset is relatively low-frequency and of limited size. Recurrent neural networks like LSTM typically require large volumes of high-resolution time series data to generalize well. In our case, the limited temporal granularity and sample size likely contributed to overfitting and poor generalization.

b) Lack of Seasonality and Complex Nonlinearity: Our preliminary analysis showed that the time series did not exhibit strong nonlinear dynamics or long-term dependencies that LSTM/RNN models are designed to capture. As a result, simpler models such as TBATS and Holt-Winters, which explicitly model trend and seasonality, outperformed neural-based models in terms of forecasting accuracy and interpretability.

c) Stability and Reproducibility: We also observed high variance in performance across different runs of LSTM and RNN, indicating instability due to the small dataset and sensitivity to initialization. This was in contrast with the more stable and reproducible results of statistical models.

These omissions should be considered when interpreting the results, as they may bias estimates of geographic and temporal risk. Future research should aim to integrate these variables to enhance model robustness and provide a more holistic understanding of crash determinants. The incorporation of high-resolution spatiotemporal data from sources like traffic sensor networks, meteorological agencies, and emergency service records represents a promising avenue for future exploration.

### Future research

To enhance the predictive capabilities of road crash models, future research should focus on incorporating a broader range of variables and exploring advanced analytical techniques. Expanding the independent variables to include detailed road conditions, infrastructure characteristics, socio-demographic and health data, and weather and environmental factors will provide a more comprehensive understanding of crash dynamics. Additionally, larger and more granular datasets may allow exploring complex machine learning models like RNNs, LSTMs, and GBMs, as well as hybrid models combining time-series analysis with causal inference or spatial analysis, will enable the capture of non-linear relationships and interactions. Disaggregated analyses focusing on state-specific trends or road-user-specific patterns will further refine our understanding of localized risks and vulnerable populations.

Furthermore, integrating real-time data, conducting comparative studies, and extending the scope of analysis to include non-fatal injuries and socio-economic consequences will significantly improve road safety research. Incorporating real-time traffic flow, weather, and incident reporting data will enable dynamic risk assessments and timely interventions. More complete data, especially the linked data of crash data reported by police and hospital registry data, is required for a deeper and more inclusive analysis^38^. Comparative studies across countries or regions with similar profiles, and longitudinal studies assessing the impact of specific interventions, will enhance the generalizability of findings and inform evidence-based policies. Finally, examining the interaction of environmental factors like weather and urban planning with crash characteristics will reveal additional pathways for improving road safety, leading to more effective and targeted interventions.

## Conclusions

This study makes a substantial contribution to the understanding and modelling of Australian road traffic fatalities (1989–2024) comparing Holt-Winters, Theta, TBATS, and VAR models. The comparative analysis clearly demonstrated that TBATS excelled in long-term forecasting (MAE: 15.11, RMSE: 18.69), effectively handling complex seasonal patterns superior to the other univariate models considered. The VAR model demonstrated competitive performance, offering unique advantages by capturing interdependence between variables and highlighting the delayed impacts of policy interventions. This study’s results are convincing due to the rigorous application of advanced time series models (TBATS, VAR) that outperform simpler alternatives (Holt-Winters, Theta) in accuracy and adaptability, as evidenced by multiple metrics and diagnostic checks. The superiority of TBATS for handling complex seasonality and VAR for capturing dynamic interdependencies was clearly demonstrated. The subsequent causal analysis using Random-effects and VAR provided statistically significant and interpretable insights consistent with road safety literature. A causal analysis, employing a random-effects model, identified key geographic, temporal, and demographic factors influencing crashes. Urbanization in major cities and challenges in remote areas significantly impacted crash rates. Temporal risks included increased accidents during summer months and nighttime driving in rural regions. Vulnerable age groups, alongside the influence of cycling infrastructure and advanced driver-assistance systems (ADAS), were also identified.

The study underscores the importance of adaptive forecasting models, particularly TBATS, whose superiority was evident in addressing dynamic road safety challenges, especially during unprecedented events like the COVID-19 pandemic. Findings offer actionable insights for policymakers, emphasizing the necessity of long-term infrastructure investments and targeted safety campaigns. The analysis provides a comprehensive understanding of crash dynamics, aiding in the development of evidence-based policies aimed at reducing road traffic fatalities in Australia.

## Supplementary Information

Supplementary Information.

## Data Availability

The data utilized for this research originates from the Australian Commonwealth Government as open-source data which available at ([https://www.bitre.gov.au/statistics/safety/fatal\_road\_crash\_database] (https://www.bitre.gov.au/statistics/safety/fatal_road_crash_database)).

## Abbreviations

AI: Artificial intelligence
AIC: Akaike Information Criterion
ADAS: Advanced driver-assistance systems
BIC: Bayesian Information Criterion
BITRE: Bureau of Infrastructure and Transport Research Economics
COVID-19: Coronavirus disease 2019
MAE: Mean absolute error
MSE: Mean squared error
NMSE: Normalized mean squared error
RMSE: Root mean squared error
TBATS: Trigonometric seasonality, Box-Cox transformation, ARMA errors, Trend and Seasonal components
VAR: Vector autoregression

## Code availability

The codes and scripts developed for this research are available at the third author’s GitHub account (https://github.com/mraminamiri/Causal-Analysis-of-Road-Fatalities).
