# Supplementary material for: From data to safer roads: predictive modelling and causal analysis of road fatalities in Australia
Source: Sci Rep. 2025 Dec 29;16:3764. doi: 10.1038/s41598-025-33744-7 (PMC12852097; doi:10.1038/s41598-025-33744-7)
Supplement: Supplementary file 1 — Supplementary Information. [file 41598_2025_33744_MOESM1_ESM.pdf]

Supplementary Material

Table A1. Dataset parameters with statistical features

Metric	count	mean	std	min	25%	50%	75%	max
Age	54499.00	40.15	21.82	0.00	22.00	35.00	56.00	101.00
Age0_16	54499.00	0.08	0.26	0.00	0.00	0.00	0.00	1.00
Age17_25	54499.00	0.26	0.44	0.00	0.00	0.00	1.00	1.00
Age26_39	54499.00	0.23	0.42	0.00	0.00	0.00	0.00	1.00
Age40_64	54499.00	0.26	0.44	0.00	0.00	0.00	1.00	1.00
Age65_74	54499.00	0.08	0.27	0.00	0.00	0.00	0.00	1.00
Age75+	54499.00	0.10	0.30	0.00	0.00	0.00	0.00	1.00
April	54499.00	0.08	0.27	0.00	0.00	0.00	0.00	1.00
August	54499.00	0.08	0.28	0.00	0.00	0.00	0.00	1.00
Crash Type_Multiple	54499.00	0.44	0.49	0.00	0.00	0.00	1.00	1.00
Crash Type_Single	54499.00	0.55	0.50	0.00	0.00	1.00	1.00	1.00
December	54499.00	0.09	0.28	0.00	0.00	0.00	0.00	1.00
February	54499.00	0.08	0.27	0.00	0.00	0.00	0.00	1.00
Gender_Female	54499.00	0.28	0.45	0.00	0.00	0.00	1.00	1.00
Gender_Male	54499.00	0.72	0.45	0.00	0.00	1.00	1.00	1.00
January	54499.00	0.08	0.28	0.00	0.00	0.00	0.00	1.00
July	54499.00	0.08	0.27	0.00	0.00	0.00	0.00	1.00
June	54499.00	0.08	0.27	0.00	0.00	0.00	0.00	1.00
MajorCities_Areas	54499.00	0.07	0.25	0.00	0.00	0.00	0.00	1.00
March	54499.00	0.09	0.29	0.00	0.00	0.00	0.00	1.00
May	54499.00	0.08	0.28	0.00	0.00	0.00	0.00	1.00
Monday	54499.00	0.12	0.32	0.00	0.00	0.00	0.00	1.00
Motorcycle_passenger	54499.00	0.01	0.08	0.00	0.00	0.00	0.00	1.00
Motorcycle_Rider	54499.00	0.13	0.34	0.00	0.00	0.00	0.00	1.00
November	54499.00	0.08	0.28	0.00	0.00	0.00	0.00	1.00
October	54499.00	0.09	0.28	0.00	0.00	0.00	0.00	1.00
Outer_Regional_Areas	54499.00	0.04	0.20	0.00	0.00	0.00	0.00	1.00
Passenger	54499.00	0.23	0.42	0.00	0.00	0.00	0.00	1.00
Pedal_Cyclist	54499.00	0.03	0.16	0.00	0.00	0.00	0.00	1.00
Pedestrian	54499.00	0.15	0.36	0.00	0.00	0.00	0.00	1.00
Period_Afternoon	54499.00	0.34	0.47	0.00	0.00	0.00	1.00	1.00
Period_Evening	54499.00	0.26	0.44	0.00	0.00	0.00	1.00	1.00
Period_Morning	54499.00	0.23	0.42	0.00	0.00	0.00	0.00	1.00
Period_Night	54499.00	0.16	0.37	0.00	0.00	0.00	0.00	1.00
Remote_Areas	54499.00	0.01	0.09	0.00	0.00	0.00	0.00	1.00
Saturday	54499.00	0.18	0.39	0.00	0.00	0.00	0.00	1.00
September	54499.00	0.08	0.27	0.00	0.00	0.00	0.00	1.00
Speed Limit	54490.00	82.98	20.68	5.00	60.00	80.00	100.00	130.00
speed_square	54490.00	7313.31	3345.33	25.00	3600.00	6400.00	10000.00	16900.00
State_ACT	56026.00	0.01	0.09	0.00	0.00	0.00	0.00	1.00
State_NSW	56026.00	0.31	0.46	0.00	0.00	0.00	1.00	1.00
State_NT	56026.00	0.03	0.17	0.00	0.00	0.00	0.00	1.00
State_Qld	56026.00	0.20	0.40	0.00	0.00	0.00	0.00	1.00
State_SA	56026.00	0.09	0.28	0.00	0.00	0.00	0.00	1.00
State_Tas	56026.00	0.03	0.17	0.00	0.00	0.00	0.00	1.00

State_Vic	56026.00	0.22	0.41	0.00	0.00	0.00	0.00	1.00
State_WA	56026.00	0.12	0.32	0.00	0.00	0.00	0.00	1.00
Sunday	54499.00	0.16	0.37	0.00	0.00	0.00	0.00	1.00
Thursday	54499.00	0.14	0.34	0.00	0.00	0.00	0.00	1.00
Tuesday	54499.00	0.12	0.32	0.00	0.00	0.00	0.00	1.00
Very_Remote_Areas	54499.00	0.01	0.09	0.00	0.00	0.00	0.00	1.00
Wednesday	54499.00	0.13	0.33	0.00	0.00	0.00	0.00	1.00
Weekend	54499.00	0.41	0.49	0.00	0.00	0.00	1.00	1.00
